# Supplementary material for: Complex‐centric proteome profiling by SEC‐SWATH‐MS
Source: Mol Syst Biol. 2019 Jan 14;15(1):e8438. doi: 10.15252/msb.20188438 (PMC6346213; doi:10.15252/msb.20188438)
Supplement: Supplementary file 6 — Dataset EV5 [file MSB-15-e8438-s006.zip › feature_plots_corum/742.pdf]

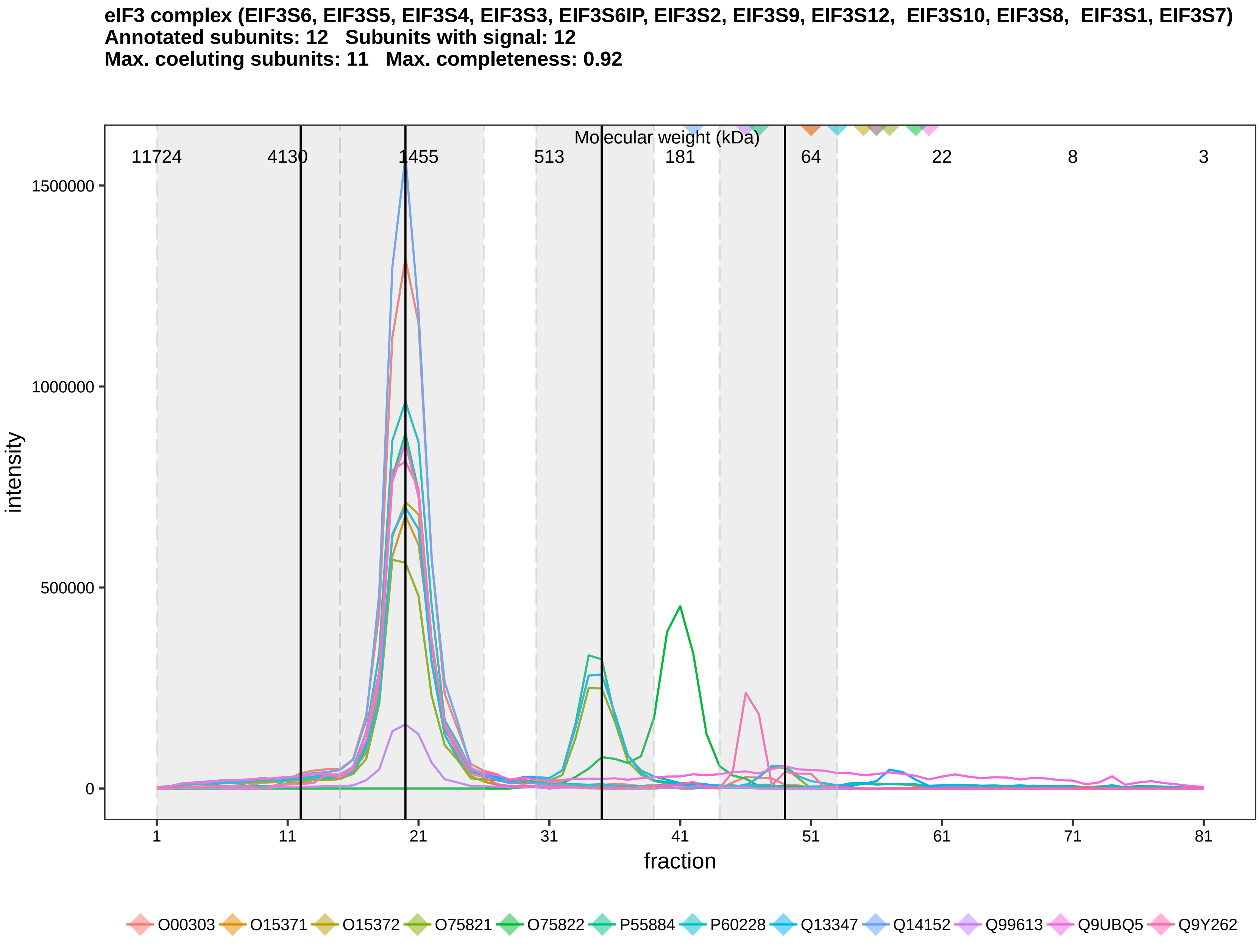

eIF3 complex (EIF3S6, EIF3S5, EIF3S4, EIF3S3, EIF3S6IP, EIF3S2, EIF3S9, EIF3S12, EIF3S10, EIF3S8, EIF3S1, EIF3S7)
Annotated subunits: 12 Subunits with signal: 12
Max. coeluting subunits: 11 Max. completeness: 0.92
Molecular weight (kDa)
11724
4130
1455
513
181
64
22
8
3
intensity
1500000
1000000
500000
0
fraction
1
11
21
31
41
51
61
71
81
O00303
O15371
O15372
O75821
O75822
P55884
P60228
Q13347
Q14152
Q99613
Q9UBQ5
Q9Y262
